# Incidence of thromboembolic complications in hospitalized COVID-19 patients in a medical ward in Japan: A single-center retrospective and prospective observational study

**DOI:** 10.1097/MD.0000000000029933

**Published:** 2022-08-19

**Authors:** Junpei Komagamine, Taku Yabuki

**Affiliations:** Department of Internal Medicine, National Hospital Organization Tochigi Medical Center, Tochigi, Japan.

**Keywords:** COVID-19, deep venous thrombosis, pulmonary embolism

## Abstract

**Background:**

A high incidence of thromboembolic complications is one of the hallmarks of COVID-19. However, there may be a difference in the incidence of thromboembolic complications between Asian and Western people. In addition, few prospective studies have been conducted to determine the incidence of thromboembolic complications in hospitalized COVID-19 patients in medical wards in Japan.

**Methods:**

A single-center retrospective and prospective cohort study was conducted to determine the incidence of thromboembolic complications in symptomatic COVID-19 patients in a medical ward in a Japanese hospital. All 1116 consecutive COVID-19 patients who were admitted to our hospital from November 1, 2020, to October 26, 2021, were included. The primary outcome was any thromboembolic complications, which included venous thromboembolism, myocardial infarction, ischemic stroke, and other arterial embolisms.

**Results:**

The median patient age was 50 (IQR, 37–61), 402 (36.0%) were women, 1005 (90.1%) were Japanese, the median body mass index was 24.1 (IQR, 21.6–27.2), and 43 (3.9%) had Padua scores of at least 4 points at admission. Regarding the severity of COVID-19, 543 (48.7%), 315 (28.2%), 204 (18.3%), and 54 (4.8%) patients had mild, moderate, severe, and critical COVID-19, respectively. Nine patients (0.8%) died, and 47 patients (4.2%) were transferred to other hospitals for intensive care. The primary outcome occurred in only 5 patients (0.5%; 95% CI, 0.1–0.8) and consisted of 3 ischemic strokes, 2 limb ischemia events, and one asymptomatic pulmonary embolism. Even in the 204 patients with severe COVID-19, the prevalence of thromboembolic complications was only 2.5% (95% CI, 0.3–4.6).

**Conclusion:**

Thromboembolic complications of COVID-19 are rare even in severe cases in a medical ward in a Japanese hospital. Further studies are needed to identify severe COVID-19 patients with a higher risk for thromboembolic complications in Japan.

## 1. Introduction

A high incidence of thromboembolic complications in hospitalized patients with coronavirus disease 2019 (COVID-19) has been reported since severe acute respiratory syndrome coronavirus 2 (SARS-CoV-2), the virus that causes COVID-19, was identified in December 2019 in China.^[1]^ A recent meta-analysis showed that the incidence of venous thromboembolism (VTE) among hospitalized COVID-19 patients was 7.1% in the ward and 27.9% in the intensive care unit (ICU).^[2]^ Another meta-analysis and systematic review reported a high incidence of arterial thromboembolic events, including myocardial infarction and ischemic stroke, in hospitalized COVID-19 patients,^[3,4]^ although the incidence of arterial thromboembolic events was lower than that of venous thromboembolic events.

These thromboembolic complications in hospitalized COVID-19 patients are associated with a worse prognosis.^[5]^ Therefore, several guidelines recommend that routine thromboprophylaxis with anticoagulants should be used for any COVID-19 patients who require hospitalization.^[6–8]^ Nonetheless, most of the studies included in the meta-analyses investigating the incidence of thrombotic complications in COVID-19 patients were conducted in Western countries.^[2–4]^ Given that the incidence of VTE in the Asian population may be lower than that in the Western population,^[9]^ it remains uncertain whether routine thromboprophylaxis with anticoagulants for any hospitalized COVID-19 patient is beneficial in the Asian population.

A recent questionnaire survey in Japan revealed that the incidence of thromboembolic complications among hospitalized symptomatic COVID-19 patients was 13.5% in patients who required ventilation or ECMO and 0.6% in those who did not.^[10]^ This implies that routine pharmacological thromboprophylaxis is not necessary for the majority of hospitalized COVID-19 patients who do not require intensive care in Japan. Nonetheless, registry data are characterized by selection bias and underreporting. Moreover, there have been no prospective studies to determine the incidence of thromboembolic complications among hospitalized COVID-19 patients in Japan. Therefore, we conducted a retrospective and prospective observational study to investigate the incidence rate among patients in a Japanese hospital.

## 2. Methods

### 2.1 Study design and setting

A retrospective (from November 2020 to March 2021) and prospective (from April 2021 to October 2021) cohort study using the electronic medical records of the National Hospital Organization Tochigi Medical Center was conducted. The National Hospital Organization Tochigi Medical Center is one of the largest general community hospitals, providing acute care for 0.5 million people in Utsunomiya, Japan. Utsunomiya is a government ordinance-designated city in Tochigi prefecture, which is located approximately 100 kilometers north of Tokyo. In addition, National Hospital Organization Tochigi Medical Center is the only hospital in the region designated to provide care for COVID-19 patients who require hospitalization but not intensive care. Utsunomiya has the policy to admit all patients with COVID-19, without specific reasons, and noncritical COVID-19 patients with higher risk factors were admitted to our hospital. During the study period, approximately one-fifth of all COVID-19 patients in this region, including asymptomatic patients, were admitted to our hospital. Because our hospital has no ICU, if the COVID-19 patients needed intensive care, they were transferred to other tertiary hospitals with ICUs. Treatment for COVID-19 in our hospital was based on the World Health Organization (WHO) guidelines.^[11]^ For VTE prophylaxis, our hospital basically adhered to the Japanese guidelines.^[12]^ Moreover, because there were no VTE events without pharmacological prophylaxis in hospitalized COVID-19 patients between February and October 2020, most of these patients, except those who required mechanical ventilation, received no pharmacological prophylaxis during their hospital stays. In addition, our hospital avoided unnecessary use of peripheral access and urinary catheters and facilitated mobilization in the room for VTE prophylaxis. This study was retrospectively registered at the University hospital Medical Information Network Clinical Trials Registry (UMIN-CTR) on November 7, 2021 (No. UMIN000046003). This research was approved by the Medical Ethical Committee of the National Hospital Organization Tochigi Medical Center (No. 2020-23) and was conducted in accordance with the principles of the Declaration of Helsinki. The need for individual informed consent was formally waived by the institutional Medical Ethics Committee because we collected de-identified data without contacting the patients. However, per the Japanese Ethical Guidelines, we displayed an opt-out statement on the webpage of the hospital to inform patients about the study and to provide the opportunity for patients to decline the use of their data.

### 2.2 The inclusion and exclusion criteria

To minimize the selection bias, all consecutive COVID-19 patients who were hospitalized at our hospital from November 1, 2020, to October 26, 2021, were included. Only patients who were symptomatic and had diagnoses confirmed by nucleic acid tests or antigen tests for SARS-CoV-2 were eligible. During the study period, 1160 patients who were clinically diagnosed with COVID-19 were admitted to our hospital. Excluding 39 asymptomatic COVID-19 patients and 5 clinically diagnosed COVID-19 patients who were not confirmed by tests for SARS-CoV-2, a total of 1116 symptomatic COVID-19 patients were included in the final analysis. There were no patients who were excluded because they declined the use of their data. The last follow-up date was October 31, 2021. The median follow-up duration was 6 days (IQR, 4–9).

### 2.3 Data collection and outcome measures

Information on patient age, sex, past medical history, clinical symptoms, regularly used medications, the presence of pneumonia, and treatment during hospitalization was retrospectively extracted from the electronic medical records. In addition, on January 1, 2019, our hospital began documenting the information needed for the Padua score^[13]^ and monitoring the incidence of VTE in all patients treated in the internal medicine ward at admission and discharge. Therefore, these data were also collected. Pneumonia was defined if the patients had new infiltration on chest images or had crackles upon physical examination. The severity of COVID-19 was defined based on WHO guidelines.^[14]^

The primary outcome was any thromboembolic complication during the follow-up period. Based on a previous study,^[5]^ thromboembolic complications included VTE, acute myocardial infarction, acute ischemic stroke, and any other arterial thromboembolisms. We included only VTEs confirmed by computed tomography or ultrasonography. In our hospital, no screening for VTE was performed for hospitalized patients, and these imaging tests were ordered only if the patient was clinically suspected of having VTE. Myocardial infarction was defined based on the fourth universal definition of myocardial infarction.^[15]^ Ischemic stroke was defined based on American guidelines.^[16]^ Other arterial thromboembolisms were included when the diagnosis was confirmed by computed tomography or ultrasonography.

### 2.4 Statistical analysis

Descriptive statistics were used to report the baseline characteristics of the included patients. For the primary outcome, the proportion of patients who had thromboembolic complications during the follow-up period was calculated with a 95% confidence interval (CI). The primary outcome was also evaluated according to the worst severity of COVID-19 or Padua score at admission (0–3 points versus more or 4 points). These analyses were performed by using Stata version 15 (LightStone, Tokyo, Japan).

## 3. Results

The median patient age was 50 (IQR 37 to 61), 402 (36.0%) were women, 1005 (90.1%) were Japanese, and the median body mass index (BMI) was 24.1 (IQR, 21.6–27.2) (Table 1; Tables 1, 2, and 3, Supplementary Digital Content, http://links.lww.com/MD/G966). Eight patients (0.7%) had active cancer, 3 (0.3%) had a past medical history of VTE, and 3 (0.3%) had trauma or surgery within one month before the index admission. Forty-three patients (3.9%) had Padua scores of at least 4 points at admission. Regarding regular medications at admission, 23 patients (2.1%) and 48 patients (4.3%) took anticoagulants and antiplatelet drugs, respectively. The median time from symptom onset was 5 days (IQR, 3–7), and 567 patients (50.8%) had pneumonia. For the worst severity of COVID-19 during hospitalization, 543 (48.7%), 315 (28.2%), 204 (18.3%), and 54 (4.8%) patients had mild, moderate, severe, and critical COVID-19, respectively. Of all patients, 47 patients (4.2%) were transferred to tertiary hospitals for intensive care, and 9 patients (0.8%) died during hospitalization. Of the 9 patients who died, the causes of death were pneumonia due to critical COVID-19 (5 patients), aspiration pneumonia (2 patients), bacteremia (1 patient), and mediastinitis (1 patient). The median duration of the hospital stay was 6 days (IQR, 3–8).

**Table 1:** Clinical and demographic characteristics of symptomatic COVID-19 patients according to disease severity*.

Characteristics	Total	Critical	Severe	Moderate	Mild
	n = 1116	n = 54	n = 204	n = 315	n = 543
Median age, y (IQR)	50 (37–61)	60 (52–74)	59 (49–71)	52 (44–62)	42 (28–54)
Women	402 (36.0)	14 (25.9)	75(36.8)	105 (33.3)	208(38.3)
Japanese	1005 (90.1)	51 (94.4)	190 (93.1)	295 (93.7)	469 (86.4)
Median body mass index, kg/m^2^ (IQR)	24.1 (21.6–27.2)	26.5 (23.8–30.9)	24.9 (22.9–27.8)	24.8 (22.2–27.4)	23.2 (20.8–26.2)
Regular medications at admission					
Antiplatelets	48 (4.3)	3 (5.6)	13 (6.4)	18 (5.7)	14 (2.6)
Anticoagulants	23 (2.1)	0 (0.0)	8 (3.9)	10 (3.2)	5 (0.9)
Venous thromboembolism risk					
Active cancer	8 (0.7)	1 (1.9)	0 (0.0)	2 (0.6)	5 (0.9)
Previous venous thromboembolism	3 (0.3)	0 (0.0)	3 (1.5)	0 (0.0)	0 (0.0)
Reduced mobility	29 (2.6)	0 (0.0)	18 (8.8)	5 (1.6)	6 (1.1)
Thrombophilic condition	0 (0.0)	0 (0.0)	0 (0.0)	0 (0.0)	0 (0.0)
Recent trauma or surgery (within 1 month)	3 (0.3)	0 (0.0)	0 (0.0)	1 (0.3)	2 (0.4)
Elderly age (≥70 years old)	178 (16.0)	18 (33.3)	58 (28.4)	54 (17.1)	48 (8.8)
Heart or respiratory failure	102 (9.1)	24 (44.4)	78 (38.2)	0 (0.0)	0 (0.0)
Acute myocardial infarction or ischemic stroke	0 (0.0)	0 (0.0)	0 (0.0)	0 (0.0)	0 (0.0)
Acute infection or rheumatologic disorder	1116 (100.0)	54 (100.0)	204 (100.0)	315 (100.0)	543 (100.0)
Obesity	138 (12.4)	15 (27.8)	35 (17.2)	34 (10.8)	54 (9.9)
Ongoing hormonal treatment	6 (0.5)	0 (0.0)	0 (0.0)	2 (0.6)	4 (0.7)
Four or more points on Padua score, n (%)	43 (3.9)	1 (1.9)	23 (11.3)	8 (2.5)	11(2.0)
Median days to admission from symptom onset (IQR)	5 (3–7)	6 (4–7)	6 (4–8)	5 (3–7)	4 (2–6)
Median duration of hospital stay, days (IQR)	6 (3–8)	5 (2–7)	10 (7–13)	6 (4–8)	4 (3–7)
In-hospital death	9 (0.8)	5 (9.3)	4 (2.0)	0 (0.0)	0 (0.0)

* The severity of COVID-19 was defined using the World Health Organization guidelines.

COVID-19, coronavirus disease 2019; IQR, interquartile range.

A total of 40 patients (3.6%) received anticoagulants during their hospital stays. Of those, 23 patients who took oral anticoagulants (6 for warfarin and 17 for direct oral anticoagulant) regularly before admission continued them during hospitalization, and 17 patients newly used anticoagulants during their hospital stays (Table 2). Of the 17 patients who newly started anticoagulants after admission, 8 patients who were intubated due to critical COVID-19 and 2 patients who had severe COVID-19 received VTE prophylaxis with unfractionated heparin during hospitalization. Three patients received direct oral anticoagulant (DOAC) for prevention of ischemic stroke for newly detected atrial fibrillation, one patient received DOAC for VTE prophylaxis for a week following surgery, and 3 patients received therapeutic-dose unfractionated heparin for pulmonary embolism and limb ischemia. No mechanical VTE prophylaxis was performed. The primary outcome occurred in 5 patients (0.5%; 95% CI, 0.1–0.8%). Six thromboembolic complications (3 ischemic strokes, 2 lower limb ischemia events, and 1 asymptomatic pulmonary embolism) occurred in these patients. All of the patients with mild and moderate COVID-19 recovered and were discharged without thromboembolic complications. Among the 204 patients with severe COVID-19, only 5 patients (2.5%; 95% CI, 0.3–4.6) had thromboembolic complications. Thromboembolic complications occurred in 1.1% and 13.0% of severe COVID-19 patients who had Padua scores of less than 4 points and 4 or more points at admission, respectively (Table 3).

**Table 2:** Prevalence of anticoagulant use and incidence of thromboembolic complications among COVID-19 patients according to disease severity*.

	Total	Critical	Severe	Moderate	Mild
	n = 1116	n = 54	n = 204	n = 315	n = 543
Anticoagulant use					
Any use	40 (3.6)	9 (16.7)†	14 (6.9)‡	11 (3.5)§	6 (1.1)‖
Regular use before admission	23 (2.1)	0 (0.0)	8 (3.9)	10 (3.2)	5 (0.9)
Newly prescribed during hospitalization	17 (1.5)	9 (16.7)†	6 (2.9)‡	1 (0.3)§	1 (0.2)‖
Thrombotic complications (primary outcome)	5 (0.4)	0 (0.0)	5 (2.5)	0 (0.0)	0 (0.0)
Deep venous thrombosis	0 (0.0)	0 (0.0)	0 (0.0)	0 (0.0)	0 (0.0)
Pulmonary embolism	1 (0.1)¶	0 (0.0)	1 (0.5)¶	0 (0.0)	0 (0.0)
Ischemic stroke	3 (0.3)	0 (0.0)	3 (1.5)	0 (0.0)	0 (0.0)
Limb ischemia	2 (0.2)	0 (0.0)	2 (1.0)‡	0 (0.0)	0 (0.0)
Myocardial infarction	0 (0.0)	0 (0.0)	0 (0.0)	0 (0.0)	0 (0.0)

COVID-19 = coronavirus disease 2019.

* The severity was defined based on the World Health Organization guidelines.

† Pharmacological prophylaxis of venous thromboembolism was started after tracheal intubation in the 8 patients. Direct oral anticoagulant was newly started because of newly-detected atrial fibrillation in one patient.

‡ One patient had a lower limb ischemia at admission. Therefore, continuous infusion of unfractionated heparin was started at admission.

§ Direct oral anticoagulant was started after admission because atrial fibrillation was newly detected at admission.

‖ Direct oral anticoagulant was prescribed for one week as surgical prophylaxis of venous thromboembolism for hip fracture by an orthopedic physician.

¶ This was asymptomatic pulmonary embolism. Thromboembolism of the left proximal artery was incidentally identified by computed tomography of the chest, which was performed to evaluate mediastinitis.

**Table 3.** The incidence of thromboembolic complications among COVID-19 patients according to the Padua score and the severity of COVID-19*.

Padua score†	Number of patients	The number of patients who had thromboembolic complications, n (%)
All COVID-19 (n = 1116)		
≥4 points	43	3 (7.0)
<4 points	1073	2 (0.2)
Critical COVID-19 (n = 54)		
≥4 points	1	0 (0.0)
<4 points	53	0 (0.0)
Severe COVID-19 (n = 204)		
≥4 points	23	3 (13.0)
<4 points	181	2 (1.1)
Moderate COVID-19 (n = 315)		
≥4 points	8	0 (0.0)
<4 points	307	0 (0.0)
Mild COVID-19 (n = 543)		
≥4 points	11	0 (0.0)
<4 points	532	0 (0.0)

COVID-19, coronavirus disease 2019.

* The severity was based on the World Health Organization guidelines.

† Padua score was used to predict the risk for venous thromboembolism in hospitalized patients. The score is calculated based on the following risk factors: active cancer (+3), previous venous thromboembolism (+3), reduced mobility (+3), known thrombophilic condition (+3), recent trauma or surgery (+2), elderly age (+1), heart or respiratory failure (+1), acute myocardial infarction or ischemic stroke (+1), acute infection or rheumatologic disorder (+1), obesity (+1), and ongoing hormonal treatment (+1). Zero to 3 points is considered a lower risk, and 4 or more points is considered a higher risk.

## 4. Discussion

Our findings showed that thromboembolic complications in the general ward were rare despite an extremely low rate of pharmacological VTE prophylaxis, even in severe COVID-19 patients, the majority of whom were East Asian people. This result is consistent with that of a recent Japanese study^[17]^ showing that only 2.2% of consecutive hospitalized COVID-19 patients in the general ward had thromboembolic complications during their hospital stays, despite a low rate of pharmacological VTE prophylaxis. These results also support the low incidence of thromboembolic complications in the Japanese COVID-19 registry^[18]^ and a previous Japanese survey.^[10]^

Despite nearly no pharmacological VTE prophylaxis, there was no occurrence of VTE in patients with mild and moderate COVID-19, and the incidence of VTE was only 2.5% in severe COVID-19 patients in the present study. This result is also similar to that of a recent Japanese study,^[17]^ which reported that almost no thromboembolic complications occurred in patients with mild and moderate severity COVID-19, but thromboembolic complications occurred in 3.0% of severe COVID-19 patients. The results of the present and recent studies^[17]^ support the Japanese COVID-19 guidelines, which recommend against pharmacological VTE prophylaxis in mild or moderate COVID-19 patients but for it in severe COVID-19 patients.^[19]^ However, thromboembolic complications occurred in only 1.1% of severe COVID-19 patients who had a Padua score of less than 4 points at admission, while 13.0% of severe COVID-19 patients who had a Padua score of 4 or more points at admission had thromboembolic complications. Moreover, although most of the beneficial effects of heparin on COVID-19 are a reduction in VTE based on recent randomized controlled trials,^[20,21]^ the prevalence of VTE among COVID-19 patients, excluding those with arterial thromboembolic complications, is much lower in Japan^[17]^ than in Western countries.^[2]^ In addition, there are several concerns about an increased risk of exposure among healthcare providers due to frequent administration of heparins, hemorrhagic complications of heparin, and heparin-induced thrombocytopenia.^[22]^ Therefore, further efforts to identify severe COVID-19 patients with a higher risk for VTE in Japan are needed.

Why is the occurrence of VTE less common among COVID-19 patients in Japan than among those in Western countries? Although one of the reasons may be underdiagnosis, it is assumed that the prevalence of hereditary thrombophilia, such as factor V Leiden, is less common in Asian populations than in Caucasian populations.^[9]^ In fact, the prevalence of the medical history of VTE among COVID-19 patients in our study and a recent Japanese study^[17]^ was much lower than that in studies conducted in Western countries.^[2]^

The strength of our study was the use of a prospective design and the largest sample size per institution to investigate the incidence of thromboembolic complications in consecutive hospitalized COVID-19 patients in the general ward. Moreover, we evaluated anticoagulant use for indications other than VTE prophylaxis during the hospital stay. Nonetheless, several limitations should be considered. First, our study was single-center research. Although the sample size per institution was large, that per study was very small. Moreover, only one-fifth of all COVID-19 patients in our region were included, which could cause a selection bias. Thus, the generalization of our results may be limited. Second, blood tests were not routinely performed in our hospital because the burden of performing these tests on nurses was reduced as much as possible. Therefore, information on blood tests for coagulopathy is not available. Third, active surveillance for VTE was not performed in our hospital. Therefore, the incidence of VTE might be underestimated. However, COVID-19 patients included in our research had a good prognosis. Given that COVID-19-related thromboembolic complications are associated with a poor prognosis,^[5]^ it is unlikely that VTEs with clinical significance was missed. Fourth, short-term follow-up missed the occurrence of VTE after hospital discharge. However, the incidence of VTE after hospital discharge is lower than that during hospitalization. Finally, the study period was quite long and included the third, fourth, and fifth epidemic waves of COVID-19 in Japan.^[23]^ Therefore, our study population included patients infected with different variants of SARS-CoV-2. Moreover, COVID-19 vaccinations were started during the study period, although most patients included in our research did not receive COVID-19 vaccination. Therefore, the change in prevalence of COVID-19 vaccination could limit the interpretation of our results. In addition, we did not collect information on the date of hospitalization of patients with COVID-19. Therefore, we could not evaluate the effect of the difference in the study period on the primary outcome.

## 5. Conclusion

Thromboembolic complications in hospitalized COVID-19 patients in the general ward are rare despite an extremely low rate of pharmacological VTE prophylaxis in Japan. Even in severe COVID-19 patients, the incidence of thromboembolic complications is much lower in Japan than in Western countries. Given the risk of pharmacological VTE prophylaxis for hemorrhagic complications and exposure of healthcare providers to infection, further strategies to identify severe COVID-19 patients with a higher risk for VTE in Japan are warranted.

## Author contributions

Conceptualization: Junpei Komagamine, Taku Yabuki.

Data curation: Junpei Komagamine.

Formal analysis: Junpei Komagamine.

Investigation: Junpei Komagamine.

Methodology: Junpei Komagamine.

Supervision: Taku Yabuki.

Validation: Junpei Komagamine, Taku Yabuki.

Visualization: Junpei Komagamine, Taku Yabuki.

Writing – original draft: Junpei Komagamine.

Writing – review & editing: Junpei Komagamine, Taku Yabuki.

## Supplementary Material


