# Supplementary material for: Incidence of thromboembolic complications in hospitalized COVID-19 patients in a medical ward in Japan: A single-center retrospective and prospective observational study
Source: Medicine (Baltimore). 2022 Aug 19;101(33):e29933. doi: 10.1097/MD.0000000000029933 (PMC9387656; doi:10.1097/MD.0000000000029933)
Supplement: Supplementary file 1 [file medi-101-e29933-s001.pdf]

Table S1. Clinical and demographic characteristics of symptomatic COVID-19 patients.

**Characteristics**	**Total (n = 1116)**
Median age (IQR), yr	50 (37–61)
Women	402 (36.0)
Ethnicity	
East Asia	1033 (92.6)
Southeast Asia	42 (3.8)
South Asia	26 (2.3)
South America	14 (1.3)
Unknown	1 (0.1)
Median body weight (IQR), kg	67 (57–77)
Median body mass index (IQR), kg/m^2	24.1 (21.6–27.2)
Residence before the index admission	
Home	1105 (99.0)
Nursing care facility or other hospital	11 (1.0)
Activities of daily living at admission	
Independent	1088 (97.5)
Partially dependent	22 (2.0)
Dependent	6 (0.5)
Vaccination for COVID-19	
None	1024 (91.8)
Once	68 (6.1)
Twice	24 (2.2)
Mean Charlson comorbidity index score (SD)	0.4 (0.8)
Current smoker	288 (25.8)
Regular alcohol drinker[a]	288 (25.9)

[a]Among 1,112 patients because data on four patients were missing.

IQR, interquartile range.

Table S2. Medical history and venous thromboembolism risk of symptomatic COVID-19 patients.

**Characteristics**	**Total (n = 1116)**
Past medical history	
Ischemic heart disease	17 (1.5)
Heart failure	11 (1.0)
Stroke	32 (2.9)
Dementia	16 (1.4)
Liver cirrhosis	3 (0.3)
Diabetes mellitus	134 (12.0)
Hypertension	306 (27.4)
Dyslipidemia	221 (19.8)
Chronic kidney disease	10 (0.9)
Dialysis	0 (0.0)
COPD/asthma	88 (7.9)
Atrial fibrillation	20 (1.8)
Median number of regular medications (IQR)	0 (0–3)
Regular medications at admission	
Immunosuppression drugs	16 (1.4)
Antiplatelets	48 (4.3)
Anticoagulants	23 (2.1)
Antipsychotics	17 (1.5)
Venous thromboembolism risk	
Active cancer	8 (0.7)
Previous venous thromboembolism	3 (0.3)
Reduced mobility	29 (2.6)
Thrombophilic condition	0 (0.0)
Recent trauma or surgery (within one month)	3 (0.3)
Elderly age (more or 70 years old)	178 (16.0)
Heart or respiratory failure	102 (9.1)
Acute myocardial infarction or ischemic stroke	0 (0.0)
Acute infection or rheumatologic disorder	1116 (100.0)
Obesity	138 (12.4)
Ongoing hormonal treatment	6 (0.5)
Padua score	
Median (IQR)	1 (1–2)

Mean (SD)	1.5 (1.0)
Four or more points, n (%)	43 (3.9)

COPD, chronic obstructive pulmonary disease; COVID-19, coronavirus disease 2019; IQR, interquartile range; SD, standard deviation.

Table S3. Clinical characteristics of symptomatic COVID-19 patients.

**Characteristics**	**Total (n = 1116)**
Median days to admission from symptom onset (IQR)	5 (3–7)
Median duration of fever from onset until discharge, days (IQR)	4 (1–8)
Symptoms at admission	
Fever	690 (61.8)
Cough	649 (58.2)
Malaise	507 (45.4)
Headache	323 (28.9)
Sore throat	301 (27.0)
Dysosmia	215 (19.3)
Dysgeusia	206 (18.5)
Arthralgia	181 (16.2)
Diarrhea	174 (15.6)
Dyspnea	165 (14.8)
Sputum	157 (14.1)
Rhinorrhea	145 (13.0)
Severity at admission	
Presymptomatic	26 (2.3)
Mild	758 (67.9)
Moderate	230 (20.6)
Severe	98 (8.8)
Critical	4 (0.4)
The worst severity during hospitalization	
Mild	543 (48.7)
Moderate	315 (28.2)
Severe	204 (18.3)
Critical	54 (4.8)
Saturated oxygen concentration at admission	
Median (IQR)	97 (96–98)
Mean (SD)	96.3 (2.3)
Oxygen therapy	
At admission	32 (2.9)
During hospitalization	220 (19.7)
Duration of oxygen therapy during hospitalization	

Median (IQR)	0 (0–0)
Mean (SD)	1.0 (3.0)
Presence of pneumonia[a]	567 (50.8)
Blood tests	123 (11.0)
Computed tomography of chest	30 (2.7)
Peripheral or central access for fluid infusion[b]	88 (7.9)
Treatment	
Remdesivir	0 (0.0)
Dexamethasone	248 (22.2)
Tocilizumab	38 (3.4)
Casirivimab/imdevimab	86 (7.7)
Duration of hospital stay, days	
Median (IQR)	6 (3–8)
Mean (SD)	6.9 (7.6)
In-hospital death	9 (0.8)
Destination from discharge	
Home	715 (64.1)
Hotel for isolation	327 (29.3)
Hospital for rehabilitation	12 (1.1)
Nursing care institution	4 (0.4)
Follow-up days from presentation	
Median (IQR)	6 (4–9)
Mean (SD)	11.5 (27.1)

[a]Defined as clinical symptoms and crackles on physical examination or infiltration on chest imaging.

[b]This excluded peripheral venous access for infusion of casirivimab/imdevimab or tocilizumab.

IQR, interquartile range; SD, standard deviation.
